# Curcumin as a regulator of Th17 cells: Unveiling the mechanisms

**DOI:** 10.1016/j.fochms.2024.100198

**Published:** 2024-03-12

**Authors:** Ehsan Ghoushi, Mohadeseh Poudineh, Negin Parsamanesh, Tannaz Jamialahmadi, Amirhossein Sahebkar

**Affiliations:** a Applied Biomedical Research Center, Mashhad University of Medical Sciences, Mashhad, Iran; b Student Research Committee, School of Medicine, Zanjan University of Medical Sciences, Zanjan, Iran; c Zanjan Metabolic Diseases Research Center, Zanjan University of Medical Sciences, Zanjan, Iran; d Department of Genetics and Molecular Medicine, School of Medicine, Zanjan University of Medical Sciences, Zanjan, Iran; e Medical Toxicology Research Center, Mashhad University of Medical Sciences, Mashhad, Iran; f Pharmaceutical Research Center, Pharmaceutical Technology Institute, Mashhad University of Medical Sciences, Mashhad, Iran; g Biotechnology Research Center, Pharmaceutical Technology Institute, Mashhad University of Medical Sciences, Mashhad, Iran

**Keywords:** Curcumin, Th17 cells, T helper 17, Autoimmune disease, Multiple Sclerosis

## Abstract

Curcumin, a polyphenol natural product derived from turmeric, possesses diverse pharmacological effects due to its interactions with various cells and molecules. Recent studies have highlighted its immunomodulatory properties, including its impact on immune cells and mediators involved in immune responses. Th17 cells play a crucial role in promoting immune responses against extracellular pathogens by recruiting neutrophils and inducing inflammation. These cells produce inflammatory cytokines such as TNF-α, IL-21, IL-17A, IL-23, IL-17F, IL-22, and IL-26. Curcumin has been shown to significantly inhibit the proliferation of Th17 cells and reduce the production of inflammatory cytokines, including TNF-α, IL-22, and IL-17. This review aims to assess the effectiveness of curcumin and its underlying mechanisms in modulating Th17 cells.

## Highlights

• Curcumin possesses diverse pharmacological effects due to its interactions with various cells and molecules.
• Th17 cells play a crucial role in promoting immune responses against extracellular pathogens.
• Curcumin can inhibit Th17 proliferation and reduce the production of inflammatory cytokines.
• This review aims to assess the effectiveness of curcumin and its underlying mechanisms in modulating Th17 cells.

## 1 Introduction

Th17 cells have been identified as a distinct lineage separate from Treg cells, Th1, and Th2. They play a crucial role in immune defense against extracellular bacteria and fungi, as well as in the pathogenesis of various autoimmune disorders (Hirota et al., 2010). The development of Th17 cells is orchestrated by a unique combination of transcriptional regulators and cytokines. TGF-β initiates the differentiation of Th17 cells in the presence of IL-21 or IL-6 (Huang et al., 2012), while IL-23 is essential for expanding the effector Th17 cell population (Langrish et al., 2005). Th17 cells produce signature cytokines such as IL-22, IL-17A, and IL-17F, which contribute to the provocation of inflammatory responses (O’Shea and Paul, 2010). Dendritic cells (DCs) serve as the primary antigen-presenting cells (APCs), bridging the gap between innate and adaptive immunity by initiating differentiation and activating unprimed T cells (Iwasaki and Medzhitov, 2010). DCs play a role in providing cytokine, antigenic, and costimulatory signals that mediate Th17 cell differentiation (Huang et al., 2012).

Curcumin, the main bioactive constituent of turmeric (Anand et al., 2008), has been used for centuries to add a specific yellow color to food and has also been utilized as a therapy in Southeastern Asia. Curcumin exhibits anticancer, anti-inflammatory, and antioxidant characteristics (Chatterjee and Pandey, 2011, Marjaneh et al., 2018, Zahedi et al., 2023, Mohajeri and Sahebkar, 2018). In the past two decades, studies have found that curcumin has immunomodulatory effects, regulating the activity of immune cells. It can reduce the production and release of several proinflammatory cytokines, such as IL-12, TNF, and IL-6, by preventing the transcription of NF-κB (Jagetia and Aggarwal, 2007). Recent reports have shown that curcumin can reduce the proliferation of alloreactive T cells and significantly suppress the differentiation of Th17 cells (Park et al., 2013). Curcumin inhibits the differentiation and development of Th17 cells through the downregulation of ORγt signaling, IL-21, and IL-6, as well as the inhibition of STAT3 phosphorylation. In summary, curcumin has the ability to hinder the differentiation and proliferation of Th17 cells by repressing the secretion of IL-23, IL-6, and IL-21. It also suppresses the production of pro-inflammatory cytokines, including IL-17 and TNF-α, in Th17 cells, thereby reducing the expression of inflammatory cells, particularly neutrophils, and resulting in decreased inflammation and infiltration (Mohammadian Haftcheshmeh et al., 2021).

Curcumin demonstrates promise in regulating T cell subsets associated with inflammatory diseases. Xiao et al. (Xiao et al., 2022) reported that curcumin downregulated pro-inflammatory Th17 cells (CD4 + CCR6 + Th17, CD4 + IL-17A + Th17, IL-17A, BATF, C-Maf, RORγt) and upregulated anti-inflammatory Treg cells (CD4 + Foxp3 + Treg, CD4 + IL-10 + Treg, IL-10, Foxp3, Eomes) in mice with diabetic colitis, suggesting its ability to restore Th17/Treg balance. Similar effects were observed in other studies of colitis (Wei et al., 2021, Guo et al., 2022) and further confirmed in a clinical trial where nano-curcumin significantly reduced serum IL-17 mRNA in patients with episodic migraine (Djalali et al., 2020). Additionally, Tahmasebi et al. demonstrated a decrease in Th17 cell number, related factors, and cytokines in both mild and severe COVID-19 patients treated with nano-curcumin (Tahmasebi et al., 2021). These findings highlight the potential of curcumin as a therapeutic agent for various inflammatory conditions by modulating T cell responses. Studies suggest nanocurcumin's ability to hinder the conversion of naive T cells into Th17 cells, potentially offering a strategy to combat Th17-driven autoimmune disorders (Dolati et al., 2018). Motivated by curcumin's potential to modulate Th17 cells, this study aimed to review curcumin’s effects on Th17/Treg balance in the context of inflammatory and autoimmune disorders.

## 2 An overview of curcumin

### 2.1 Chemical properties of curcumin

The main active compound in turmeric is curcumin (C21H20O6), which gives turmeric its yellow color. Curcumin was first discovered by Lampe and Milobedeska in 1910 as a diferuloylmethane, specifically 1,7-bis(4-hydroxy-3-methoxyphenyl)-1,6-heptadiene-3,5-dione. It has a molecular weight of 368.7 (Jacob et al., 2008). Curcumin is a bis-α-β-unsaturated β-diketone and is insoluble in water. The predominant form of curcumin is the bis-keto form, but at a pH higher than 8, the enolate form becomes the predominant form (Sharma et al., 2005). This means that at a basic pH, curcumin acts as an electron donor, while at pH 3–7, it exhibits significant hydrogen atom donor function (Jovanovic et al., 2001). However, curcumin is unstable and degrades within thirty minutes at basic pH. Antioxidants such as ascorbic acid or human blood can inhibit this degradation (Wang et al., 1997).

### 2.2 Pharmacokinetic and pharmacodynamic properties of curcumin

Metabolism, absorption, and tissue distribution are crucial factors in assessing a compound's viability as a treatment. Several animal studies have shown that curcumin has limited systemic bioavailability due to its rapid metabolism and conjugation in the liver, followed by excretion in fecal matter (Ireson et al., 2001). Human studies on curcumin's systemic bioavailability, metabolites, and pharmacokinetics have primarily focused on patients with cancer. A phase I clinical trial investigating the effects of curcumin on multiple precancerous lesions revealed poor systemic bioavailability and absorption of curcumin (Hsieh, 2001). Another phase I trial examining the impact of curcumin on advanced colorectal cancer did not detect curcumin concentration in the plasma at lower doses. However, sulfate and glucuronide metabolites of curcumin were identified in plasma at higher doses (Sharma et al., 2004). In contrast, Garcea et al. (Garcea et al., 2005) found that patients with different stages of colorectal cancer who took 3.6 g of curcumin for 7 days had pharmacologically effective concentrations of curcumin in both normal and colorectal tissue (Menozzi et al., 2009). The therapeutic efficacy of curcumin is limited due to its rapid plasma clearance and conjugation. Therefore, complexing curcumin with other compounds to enhance systemic bioavailability is a useful approach. The addition of an adjuvant can prevent curcumin metabolism, making it of great interest to combine adjuvants with curcumin. Combining piperine with curcumin has been shown to increase curcumin bioavailability in both rats and humans (Shoba et al., 1998). Additionally, combining curcumin with micelles and phospholipid complexes (Li et al., 2005, Maiti et al., 2007), liposomes (Li et al., 2005), and nanoparticles (Bisht et al., 2007) can increase curcumin bioavailability by improving cellular permeability, providing long-term circulation, and enhancing resistance to metabolic processes (Xie et al., 2011).

### 2.3 Curcumin’s biological activities

Curcumin has several roles in the regulation of enzymes involved in inflammation, adhesion molecules, transcription factors, protein kinases, cytokines, and redox balance. It possesses various pharmacological functions, including antimicrobial, antitumor, anti-inflammatory, antiviral, antioxidant, and antifungal properties (Howells et al., 2021, Heidari et al., 2023, Bagheri et al., 2020, Cicero et al., 2020, Keihanian et al., 2018, Khayatan et al., 2022, Mokhtari-Zaer et al., 2018, Panahi et al., 2019, Sahebkar, 2010, Sahebkar, 2014, Iranshahi et al., 2010, Mohammadi et al., 2019, Panahi et al., 2012). It also plays a protective role against autoimmune diseases.

Diseases such as psoriasis (Kurd et al., 2008, Buonocore et al., 2010); inflammatory bowel disease (IBD) (Epstein et al., 2010), rheumatoid arthritis (RA) (Park et al., 2007), pancreatitis, and some autoimmune models (Hsu and Cheng, 2007), osteoarthritis (Csaki et al., 2009), autoimmune encephalomyelitis (Xie et al., 2009), Alzheimer's disease (Baum et al., 2008), as well as the prevention of diabetes and atherosclerosis (Wongcharoen and Phrommintikul, 2009, Jain et al., 2009) — all of which are regarded as Th17-mediated autoimmune diseases — are treated with curcumin.

Several studies have demonstrated that curcumin can enhance the antitumor effects of chemotherapeutics, including paclitaxel, against various cancers while also reducing the side effects of conventional chemotherapies (Ashrafizadeh et al., 2020). Curcumin acts as an active scavenger of reactive oxygen species (ROS) and reactive nitrogen species. Moreover, it increases the synthesis of glutathione (GSH), a crucial intracellular antioxidant (Menon and Sudheer, 2007, Zheng et al., 2017). Curcumin also plays a role in protecting against catalase activities and reducing the levels of glutathione S-transferases (GST), superoxide dismutase (SOD), and glutathione peroxidase (GPx) (Arshad et al., 2017). Because oxidative stress is closely related to inflammation, it indirectly inhibits pro-inflammatory reactions in the immune system (He et al., 2015). Notably, curcumin is a potent immunomodulator, with strong interactions with different types of immune cells, including neutrophils, T cells, mast cells, epithelial cells, eosinophils, and basophils, resulting in the regulation of pathological immune system reactions (Panahi et al., 2015, Mohammadi et al., 2022, Rahimi et al., 2019). Curcumin can also interact with different transcription molecules and signaling factors, such as Janus kinases/Signal transducer and activator of transcription (JAKs/STATs), mitogen-activated protein kinases (MAPKs), and nuclear factor-κB (NF-κB), which mediate its immunomodulatory properties (Zhao et al., 2016, Pan et al., 2013).

## 3 Th17-mediated immune responses.

T helper 17 (Th17) cells are a subset of CD4 + T cells that play a critical role in immune defense by producing cytokines, such as interleukin 22 (IL-22), interleukin 17 (IL-17), and interleukin 25 (IL-25). These cytokines recruit additional immune cells to the site of infection and activate innate immune responses (Miossec and Kolls, 2012). However, dysregulation of Th17-mediated immune responses can lead to various autoimmune and inflammatory diseases, highlighting the significance of tightly regulating the Th17 cell response (Miossec and Kolls, 2012, Haftcheshmeh et al., 2022). On the other hand, regulatory T (Treg) cells are a subset of T cells that suppress immune responses to self-antigens and prevent autoimmune reactions (Miossec and Kolls, 2012, Haftcheshmeh et al., 2022). CD4 + CD25 + FOXP3 + regulatory T cells are an essential component of the immune system that helps maintain immune tolerance (Miossec and Kolls, 2012, Haftcheshmeh et al., 2022). These T cells can produce anti-inflammatory cytokines, including IL-10 and TGF-β, which help suppress immune responses and prevent the development of autoimmune diseases and allergies (Miossec and Kolls, 2012, Haftcheshmeh et al., 2022). By regulating the immune system in this way, Tregs can attenuate allergic reactions and prevent tissue damage caused by inflammation (Haftcheshmeh et al., 2022, Akdis et al., 2005, Boonpiyathad et al., 2020). The balance between Th17 and Treg cells is crucial for a healthy immune response, and imbalances in this equilibrium can lead to chronic inflammatory disorders (Haftcheshmeh et al., 2022, Akdis et al., 2005, Boonpiyathad et al., 2020). Therefore, understanding the mechanisms that regulate Th17/Treg-mediated immune responses is crucial for developing new therapies for autoimmune and inflammatory diseases (Miossec and Kolls, 2012, Haftcheshmeh et al., 2022, Akdis et al., 2005, Boonpiyathad et al., 2020).

## 4 Immunomodulatory effects of curcumin

Curcumin, a natural compound found in turmeric spice, has been revealed to possess potent immunomodulatory effects (Haftcheshmeh et al., 2022). The immunomodulatory effects of curcumin, which confer protection against immune-mediated diseases, are attributed to its ability to interact with cells of the immune system (Gao et al., 2004, Gautam et al., 2007, Momtazi-Borojeni et al., 2018). Curcumin can influence both the innate and adaptive immune reactions (Haftcheshmeh et al., 2022, Gao et al., 2004). Curcumin's immunomodulatory properties arise from its ability to modulate the expression of numerous pro-inflammatory chemokines and cytokines, including interleukin (IL)-1β, tumor necrosis factor (TNF)-α, IL-6, and monocyte chemoattractant protein-1 (MCP-1) (Haftcheshmeh et al., 2022, Gao et al., 2004, Momtazi-Borojeni et al., 2018, Panahi et al., 2016). These cytokines are crucial in the initiation and maintenance of the inflammatory reaction, and the modulation of their expression by curcumin can effectively suppress inflammation (Gao et al., 2004, Momtazi-Borojeni et al., 2018). Furthermore, curcumin has been shown to have an impact on the immune cell populations themselves (Ahmed et al., 2015, Cundell and D, Wilkinson F. , 2014, Srivastava et al., 2011). Studies have shown that curcumin can increase the activity of natural killer (NK) cells, which play a critical role in the innate immune response against viruses and cancer cells (Ahmed et al., 2015, Cundell and D, Wilkinson F. , 2014, Srivastava et al., 2011, Mohammadi et al., 2019, Zhou et al., 2011). Curcumin has also been found to enhance the proliferation and activity of T cells, which are vital in the adaptive immune reaction against pathogens. Moreover, curcumin's immunomodulatory effects extend to the regulation of immune cell signaling pathways (Rahimi et al., 2019, Ahmed et al., 2015, Cundell and D, Wilkinson F. , 2014, Srivastava et al., 2011, Mohammadi et al., 2019, Zhou et al., 2011). Curcumin has been found to inhibit the activation of the nuclear factor-kappa B (NF-κB) pathway, a critical pathway involved in the expression of pro-inflammatory cytokines (Rahimi et al., 2019, Ahmed et al., 2015, Cundell and D, Wilkinson F. , 2014, Zhou et al., 2011, Mohammadi et al., 2017, Mohammadi et al., 2021).

In conclusion, curcumin's immunomodulatory effects make it a promising therapeutic agent for various inflammatory and autoimmune disorders (Rahimi et al., 2019). The compound's ability to suppress inflammation, enhance immune cell activity, and regulate immune cell signaling pathways suggests its potential as an alternative or complementary treatment option (Rahimi et al., 2019, Zhou et al., 2011, Mohammadi et al., 2017). Nonetheless, additional study is required to define the full extent of curcumin's immunomodulatory effects and its optimal dosage and administration for therapeutic purposes.

## 5 Immunomodulatory effects of curcumin on Th17-mediated immune reactions

### 5.1 Immunomodulatory effects of curcumin in human studies.

Recent research has focused on the ability of curcumin to modulate Th17-mediated immune responses, which play a critical role in the development of several autoimmune diseases (Rahimi et al., 2019). It has been revealed that curcumin is capable of inhibiting the growth and development of Th17 cells and can also decrease the production of certain pro-inflammatory cytokines, including IL-17, IL-6, and tumor necrosis factor-alpha (TNF-α) (Rahimi et al., 2019, Brück et al., 2017). Additionally, curcumin has been found to increase the production of regulatory T cells (Tregs), which help suppress excessive immune reactions and maintain immune homeostasis (Gao et al., 2004). These immunomodulatory effects of curcumin have been attributed to its ability to prevent various signaling pathways involved in Th17 cell differentiation and activation, including the STAT3, MAPK, and NF-κB pathways (Rahimi et al., 2019, Gao et al., 2004).

Investigators are studying the possibility of identifying small molecules that could target and modify the function of Th17 cells or their downstream signaling pathways, with the aim of preventing the master regulator of Th17 cells, RORγt, as a potential therapeutic strategy for inflammatory and autoimmune disorders (Rahimi et al., 2019, Isono et al., 2014). Recent studies have revealed the role of Th17 cells in neuroinflammatory disorders like AD, MS, PD, and schizophrenia (Tahmasebinia and Pourgholaminejad, 2017). The cytokines expression associated with Th17 cells, IL-17A and RORγt, has been significantly reduced in groups treated with curcumin, indicating that curcumin can prevent the induction, differentiation, development, and progression of Th17 cells in disorders related to Th17 cells (Tahmasebinia and Pourgholaminejad, 2017).

Curcumin has been shown to have a positive effect on Treg cell populations and function, suggesting its potential use as an immunotherapeutic treatment for Treg-mediated diseases such as tumors and sepsis (Rahimi et al., 2019). The study suggests that curcumin acts as an immunosuppressive and immunomodulatory agent by inhibiting cell-mediated cytotoxicity (CMC), cell growth, and the production of pro-inflammatory cytokines. This is achieved by preventing the activation and nuclear translocation of NF-kB (Rahimi et al., 2019).

Based on the placebo-controlled trial conducted on 89 patients with UC, Hanai et al. (Hanai et al., 2006) reported that curcumin had better clinical efficacy and was better tolerated compared to those who were taking a placebo plus SZ or mesalamine. Furthermore, in an additional study, Skyvalidas et al. (Skyvalidas et al., 2020) studied the role of curcumin as a dietary immunosuppressant in psoriatic disease and reported that curcumin may be effective due to its ability to prevent the production of pro-inflammatory cytokines, IL-17, and IFN-γ.

### 5.2 Immunomodulatory effects of curcumin in animal studies.

Studies have revealed that curcumin can inhibit the differentiation and proliferation of Th17 cells, as well as decrease the secretion of pro-inflammatory cytokines such as IL-17, IL-6, and tumor necrosis factor-alpha (TNF-α) in mice (46, 64). According to a study by Zhao et al. (Zhao et al., 2012), taking curcumin supplements can prevent the suppression of CD4 + CD25 + Treg cells in mice. Based on the research conducted by Brewer et al. (Brewer et al., 1999), the two distinct subsets of T cells - regulatory T cells (Tregs) and T-helper17 (Th17) cells - have opposite functions in vivo than Th1 and Th2 cells. Tregs play a vital role in regulating the immune reaction by reducing inflammation and allergic reactions through their inhibitory acts (Ma et al., 2013). In a murine model of asthma, Dongaci et al. (Doganci et al., 2005) demonstrated that lower levels of Tregs led to improved airway hyperreactivity, thus indicating a link between Tregs and airway physiology (Seroogy and Gern, 2005).

Boosting Treg function in patients could also be a promising therapeutic approach for managing allergic diseases, including asthma, as Tregs can effectively inhibit disease-inducing immune responses (Ma et al., 2013). Th17 cells and their cytokines, specifically IL-17A and IL-17F, have a role in the recruitment of neutrophils in response to antigens in asthma models using mice (Wakashin et al., 2008). Moreover, they enhance the recruitment of eosinophils into the airways, which is mediated by Th2 cells (Wakashin et al., 2008). The data from the study conducted by Ma et al. (Ma et al., 2013) indicates that curcumin has a significant impact on cytokine levels, with a reduction in IL-17A and an increase in IL-10. Additionally, the study revealed that curcumin had a notable effect on immune cells, with a decrease in Th17 cells and an increase in Tregs (Ma et al., 2013).

Based on the study by Lee et al. (Lee et al., 2017), the administration of a diet rich in curcumin was found to reduce the effects of immune disorder induced by scurfy, which is a model of Immunodysregulation polyendocrinopathy enteropathy X-linked syndrome (IPEX), in mice. This was achieved by preventing the responses of Th1, Th2, and Th17 cells (Lee et al., 2017).

### 5.3 Toxicity of curcumin

Turmeric contains a natural compound called curcumin, which has undergone thorough research due to its potential health advantages (McGilligan et al., 2007). Despite being considered generally safe, the use of high doses of curcumin over a prolonged duration or in excessive quantities can pose toxicity risks (McGilligan et al., 2007). Research conducted by McGilligan et al. (McGilligan et al., 2007) revealed that rats experienced liver toxicity when given high doses of curcumin (exceeding 1000 mg/kg body weight/day). Nonetheless, the study noted that lower dosages (up to 100 mg/kg body weight/day) did not result in any substantial toxicity (McGilligan et al., 2007). Although in vitro research has indicated that curcumin might have the potential to cause damage to DNA (genotoxic effects), it should be noted that such findings have not been replicated in vivo (Shanmugam et al., 2015).

In general, the available evidence indicates that moderate consumption of curcumin, as part of a balanced diet, poses no significant risk. Nonetheless, individuals should be careful when using high doses or when taking curcumin in conjunction with medications.

### 5.4 Adverse effect of curcumin

Consuming large amounts of curcumin can result in gastrointestinal issues like diarrhea and nausea (Jurenka, 2009). While one study discovered that humans were able to tolerate doses of up to 8 g per day without any issues, exceeding this dosage could result in negative effects (Jurenka, 2009). The consumption of curcumin could potentially impede the absorption and metabolism of certain medications (Hsieh, 2001). For instance, it may intensify the blood-thinning properties of warfarin, which may result in bleeding. Additionally, it might have an impact on chemotherapy drugs, diminishing their efficacy (Hsieh, 2001).

It's worth mentioning that most of the adverse effects associated with curcumin have been observed in studies that involved high doses of curcumin supplements. Therefore, incorporating moderate amounts of turmeric and curcumin in the regular diet is generally considered safe and healthy.

## 6 The effect of Curcumin as an epigenetic regulator on Th17 and Treg plasticity

Research has confirmed that Th17 cells can transform into other types of CD4 T cells, showcasing their remarkable adaptability (Obermajer et al., 2014). For instance, it has been shown that some cells co-express RORγt (Th17 marker) and Foxp3 (Treg marker), suggesting a transient immunosuppressive stage with initial IL-17 expression during Th17-to-Treg conversion (Obermajer et al., 2014).

Studies by Yang et al. (Yang et al., 2008) and Ye et al. (Ye et al., 2011) provide evidence for the conversion of Th17 cells into Tregs in vitro. While TGF-β alone promotes Treg development, adding IL-6, IL-1, or IL-23 alongside it reverses this effect, suppressing Foxp3 and driving IL-17 production (Yang et al., 2008, Ye et al., 2011, Peng et al., 2007). Notably, experiments by Ye et al.'s with tumor-infiltrating lymphocytes showcase a significant shift towards FOXP3 + Tregs and reduced IL-17 + Th17 cells with TCR stimulation, highlighting the potential for Th17 to Treg conversion under specific conditions (Ye et al., 2011).

Biopsy samples collected from patients with B-Cell Non-Hodgkin's lymphoma demonstrate that malignant B cells stimulate the expression of Foxp3, facilitate the development of regulatory T cells (Treg), and consequently suppress the differentiation of Th17 cells. As a result, a profoundly inhibitory tumor microenvironment is established (Yang et al., 2009). This indicates that there exists a close association between Th17 and Treg within the tumor microenvironment. In mice with tumors, it has been observed that Th17 cells serve as a source for tumor-induced Treg cells (Downs-Canner et al., 2017). Stephanie Downs-Canner et al. have put forth the notion that the transdifferentiation of Th17 cells plays a significant role in the emergence of Treg cells within the tumor microenvironment. Recent literature has brought to light that HIF-1α directly induces RORγt transcription, thereby regulating the downstream Th17 genes and facilitating Th17 differentiation. Furthermore, Eric V Dang has made a noteworthy observation that Foxp3 is highly responsive to hypoxia, and HIF-1α impedes Treg differentiation through the glycolytic pathway, thereby enabling the degradation of Foxp3 protein (Dang et al., 2011, Shi et al., 2011).

As recent research suggests that tumor-infiltrating Th17 cells can convert into immunosuppressive Tregs. This transformation is dependent on alterations in their epigenetic landscape and gene expression patterns, encompassing key transcriptional factors and cytokine genes (Ye et al., 2011, Yang et al., 2009). Metabolic pathways might regulate this plasticity, potentially influencing the overall prevalence of Th17 cells. A compelling example comes from Xu et al., who demonstrated that manipulating a single step in the glutamate pathway could reprogram Th17 cells into Tregs. They achieved this by reducing 2-hydroxyglutarate levels, leading to decreased methylation of the Foxp3 promoter, ultimately triggering Foxp3 expression and silencing the Th17 master regulator RORγt (Marshall et al., 2014). This remarkable finding highlights the potential for metabolic interventions to influence Th17/Treg balance, offering exciting avenues for therapeutic strategies aimed at boosting anti-tumor immunity. In gastrointestinal cancers, imbalances in gut microbia (dysbiosis) are increasingly recognized as potential environmental factors influencing cancer development through epigenetic changes within the host (van der Veeken et al., 2020). Dietary metabolites originating from the gut microbiota have the potential to serve as crucial regulators of epigenetic enzymes. Commensal bacteria produce short-chain fatty acids that can block an enzyme called histone deacetylases, leading to increased expression of histone acetylation of the Foxp3 locus, resulting in an increase in its expression and the differentiation of Treg cells (Lio and Hsieh, 2008).

The recent discovery of Th17 cell conversion into immunosuppressive Tregs *via* epigenetic changes (Ye et al., 2011, Yang et al., 2009) sheds light on fascinating possibilities for curcumin's potential role in regulating Th17 balance. As highlighted in the previous paragraph, dietary metabolites, like short-chain fatty acids, can modify gene expression through epigenetic enzymes, influencing Treg differentiation (Lio and Hsieh, 2008). Similarly, curcumin, a dietary compound known for its diverse biological effects, exhibits promising epigenetic modulatory properties. Studies suggest curcumin can inhibit enzymes like DNA methyltransferases and histone deacetylases, potentially influencing gene expression patterns related to Th17 differentiation and function (Kaplan et al., 2015).

Dietary components like curcumin can influence gene expression through epigenetic modifications, which regulate gene activity without altering the DNA sequence itself. These modifications encompass DNA methylation, histone modifications, and microRNA expression levels (Zhu et al., 2014, Azad and Tomar, 2015, Boyanapalli and Kong, 2015, Hassan and Rehman MS-u, Khan MS, Ali MA, Javed A, Nawaz A, , 2019;10:514.). Studies have specifically demonstrated that curcumin treatment leads to a downregulation of several histone deacetylases (HDACs), including HDAC1, HDAC3, and HDAC8. This, in turn, results in significantly increased levels of histone H4 acetylation, suggesting enhanced gene accessibility and potential transcriptional activation. Western blot analysis further confirms a dose-dependent decrease in protein levels of the aforementioned HDACs upon curcumin exposure (Liu et al., 2005). While the precise molecular mechanisms underpinning curcumin's HDAC inhibitory action remain partially elucidated, evidence suggests modulation of HDAC expression as a potential contributor. One study reports a curcumin-induced decrease in HDAC2 expression in monocytes, supporting this hypothesis. Further research is necessary to fully delineate the intricate signaling pathways and targets involved in curcumin's epigenetic modulatory effects on Th17 conversion (Meja et al., 2008).

Treatment with curcumin has been shown to increase phosphorylation of histone H3 at the Ser10 residue in mouse models, indicating increased transcriptional activity (Tikoo et al., 2008). Additionally, curcumin appears to downregulate Aurora A gene expression in human bladder cancer cells, which likely contributes to the observed reduction in phosphorylated histone H3 at Ser10 (Liu et al., 2011). These findings suggest that curcumin may influence the activity of histone kinases and phosphatases, affecting gene expression patterns (Azad et al., 2013). Furthermore, evidence indicates that curcumin can modulate diverse signaling pathways, including MAPK, Akt, p53, androgen receptor, Ras, and estrogen receptor, in various human cell lines (Ranjan et al., 2004, Wu et al., 2003), potentially contributing to its diverse biological effects.

Studies have shown that curcumin can inhibit an enzyme called DNMT1, leading to decreased methylation of certain genes in various human cell lines (Jha et al., 2010, Kuck et al., 2010, Liu et al., 2009). This inhibitory effect seems to involve a direct interaction between curcumin and the enzyme's active site (Liu et al., 2009). Interestingly, even low concentrations of curcumin have been shown to reduce overall DNA methylation levels in leukemia cells (Khor et al., 2011). Further, curcumin can directly inhibit the activity of similar enzymes in test tube experiments (Khor et al., 2011). While genome-wide studies suggest that curcumin primarily affects only partially methylated genes in colorectal cancer cells (Link et al. (2):e57709.), it can also specifically target the hypermethylation of a gene called FANCF, leading to increased expression of this gene in certain cancer cells (Shu et al., 2011). The ability of curcumin to influence DNA methylation patterns across various cell types, as demonstrated by its interaction with DNMT1 and other similar enzymes (Jha et al., 2010, Kuck et al., 2010, Liu et al., 2009, Khor et al., 2011, Link et al., 2013, Shu et al., 2011), opens up exciting possibilities for understanding its potential effects on Th17 cells. iven curcumin's ability to decrease overall DNA methylation and target specific hypermethylated genes, it's possible that it could influence the epigenetic landscape of Th17 cells.

This raises the intriguing possibility that curcumin could interact with the Th17/Treg conversion pathway, either promoting Treg development or suppressing Th17 activity through its epigenetic effects. Further research exploring this link could pave the way for novel therapeutic strategies utilizing curcumin's unique epigenetic potential to modulate immune responses and potentially combat Th17-associated diseases.

## 7 The Effect of curcumin on Th17 meditated by DCs.

Dendritic cells (DCs) play a crucial role in initiating and directing T-cell responses, including the development of Th17 cells (Iwasaki and Medzhitov, 2010, Joffre et al., 2009). Th17 cells rely on specific cytokines – IL-6, IL-21, and IL-23 – for differentiation and maintenance, with DCs being key producers of IL-6 and IL-23 (Huang et al., 2012). Interestingly, studies suggest that curcumin, a natural compound, can impact Th17 development by modifying DC function (Zhao et al., 2017, Kim et al., 2005). Findings by Zhao et al. (Zhao et al., 2017) indicate that curcumin reduces IL-6 and IL-23 expression in DCs, potentially hindering both the initial and later stages of Th17 differentiation. Moreover, Gi-Young Kim et al. (Kim et al., 2005) observed reduced production of pro-inflammatory cytokines (IL-1, IL-6, and TNF-α) in curcumin-treated DCs, further suggesting its potential immunomodulatory effects. While the impact of curcumin on IL-23 expression in DCs has received less attention, the existing evidence highlights its potential to influence Th17 development through regulating DC-derived cytokine production. Further research is warranted to fully understand the precise mechanisms and therapeutic implications of curcumin in this context.

## 8 The effect of curcumin on Th17 in autoimmune diseases.

Autoimmune diseases are the result of immunological tolerance to self-antigens. The mechanism that leads to poor self-tolerance in autoimmune diseases is still unknown. One possible mechanism is described as the deficits in migratory, functional, and numerical characteristics of Treg cells. Curcumin can shift the balance of Th1/Th17 towards Th2/Treg and induce the B cells' differentiation into a subset of B10 cells. Curcumin can inhibit the Th1/Th2/Th17 reactions resulting in improvement of scurfy-induced immune disorder (Lee et al., 2017).

### 8.1 Psoriasis

Psoriasis, an inflammatory skin disease, involves both immune cell infiltration and excessive keratinocyte growth (Schön, 2005, Lowes et al., 2007). Recent research highlights the critical role of the IL-17A/IL-23 cytokine axis in its development (Di Cesare et al., 2009, Zaba et al., 2009). Studies suggest it can suppress pro-inflammatory cytokines like IL-1β and IL-6, potentially leading to downregulated IL-23 and IL-17A expression (Sun et al., 2013, Sun et al., 2015). Supporting this, a mouse model study reported a 50 % reduction in Th17 cell proliferation and a 60 % decrease in inflammatory cytokines like IFN-γ, IL-23, TNF-α, and IL-22 upon curcumin treatment (Kang et al., 2016).

### 8.2 Rheumatoid arthritis

Several studies have demonstrated the role of IL-17A and IL-17F in inducing and exacerbating inflammation in RA (Van Den Berg and Miossec, 2009, Yao et al., 1950). Elevated levels of IL-17 in the RA synovial fluid suggest its potential role in the development of RA (Chabaud et al., 1999). Research on mice treated with IL-17R or Th17-deficient mice indicates a significant decrease in RA symptoms (Nakae et al., 2003). Furthermore, Th17 cells contribute to leukocyte infiltration, particularly neutrophils, in inflamed joints and activate tissue-degrading mediators such as matrix metalloproteinases (MMPs), elastase, and collagenases, leading to joint destruction (Chabaud et al., 1999, Miossec, 2003). Additionally, IL-17 dysregulates chondrocyte metabolism, contributing to the degradation of the cartilage matrix (Benderdour et al., 2002).

IL-17 stimulates the increased production of nitric oxide and prostaglandin E2 (PGE2) by chondrocytes (Attur et al., 1997, LeGrand et al., 2001). TNF-α and IL-17A together activate synovial fibroblast cells to express IL-8 and IL-6 in the synovial fluid, which further contributes to joint inflammation. IL-17 also inhibits collagen remodeling (Chabaud et al., 2001).

Studies by Kanakasabai et al. and Okamoto et al. demonstrated that curcumin can inhibit the production of IL-17 and IL-23 in collagen-induced arthritis (CIA) and experimental autoimmune encephalomyelitis (EAE) in mice, respectively, resulting in reduced clinical symptoms of the diseases (Kanakasabai et al., 2012, Okamoto et al., 2011). Furthermore, curcumin can reduce the differentiation of naïve CD4 + T cells into Th17 cells, thereby inhibiting Th17-induced inflammation and ameliorating autoimmune-related disorders (Dolati et al., 2018, Han et al., 2014, Handono et al., 2015). Multiple studies indicate that curcumin can block the proliferation and differentiation of Th17 cells by down-regulating the expression of RORγt, IL-21, and IL-6 (Xie et al., 2009, Kanakasabai et al., 2012) (Table 1).

**Table 1:** The effect of curcumin on Th17 in autoimmune and inflammatory diseases.

Author, Year	Type of disease	Cucumin Dose	Study Design	Model	Decreased the function and frequency of Th17 cells	Elevated the function and frequency of Tregs	Immunologic Effects
Kang et al., 2016	Psoriasis	40 mg/Kg	In vitro and in vivo	Mice	√		The proliferation of Th17 inhibited, thereby reduced the expression of TNF-α, IFN-γ, IL-22, and IL-23
Kanakasabai et al., 2012	Autoimmune Encephalomyelitis	0 or 100 μg	In vitro and in vivo	C57BL/6 mice	√	√	Th17 and Th1 cell responses was inhibited mediated by by APC-derived IL-12 family cytokines
Dolati et al., 2018	Multiple Sclerosis	80 mg/day	Clinical trial	Patients with mutiple sclerosis	√		The transcriptional activity of RoRγ T was inhibited, thereby inhibiting the expression of IL17 and IL-23R genes
Han et al. (Han et al., 2014); 2014	Autoimmune Neuritis	100 mg/ kg/day	In vitro and in vivo	Rat	√	√	The transcriptional activity of RoRγ T was inhibited, thereby inhibiting the expression of IL6.
Handono et al., 2015	systemic lupus erythematosus	0.1 and 1 µg/ml	In vitro	systemic lupus erythematosus patients	√	√	The differentiation and function of Th17 cells were inhibited by JAK3/ STAT3 pathway
Xie et al., 2009	autoimmune encephalomyelitis	100 or 200 mg/kg/day	In vitro and in vivo	Rat	√		Th17 cytokine expression was inhibited by affecting STAT3 phosphorylation and RORγ T expression
Ramezani et al. (Ramezani et al., 2022); 2022	collagen-induced arthritis	200 mg/kg	In vivo	Rat	√		Th17 was inhibited, thereby reduced the expression of TNF-α, IL-17A, and MMp-8
ELBini-Dhouib et al., 2022	Multiple sclerosis	100 mg/kg	In vitro and in vivo	C57BL/6 mice	√		Th17 was inhibited, thereby reduced the expression of TNF-α, IFN-γ, IL-2, and IL-6
Guo et al., 2022	Ulcerative colitis	NA	In vivo	Rat	√	√	Th17 was inhibited, thereby reduced the expression of IL-17A
Wei et al., 2021	DSS-induced colitis	100 mg/kg/day	In vivo	BALB/c male mice	√	√	Th17 was inhibited, thereby reduced the expression of IL6-, IL-17, and IL-23
Xiao et al. (Xiao et al., 2022); 2022	Diabetes-induced Colitis	100 mg/kg/day	In vivo	C57BLKS/J mice	√	√	The transcriptional activity of RoRγ T was inhibited, while FoxP3 mRNA level was increased, regulating Th17/Treg balance
Ma et al., 2013	Asthma	50 mg/kg, 100 mg/kg, 200 mg/kg	In vivo	female BALB/c mice	√	√	Th17 was inhibited, thereby reduced the expression of IL-17 and IL-10
Brück et al., 2017	Autoimmune encephalomyelitis	80.5 mg/day	In vitro and in vivo	SJL mice	√		Th17 and Th1 cell differentiation is inhibited by inhibition of JAK2/STAT3 signaling pathwa

**Fig. 1:**
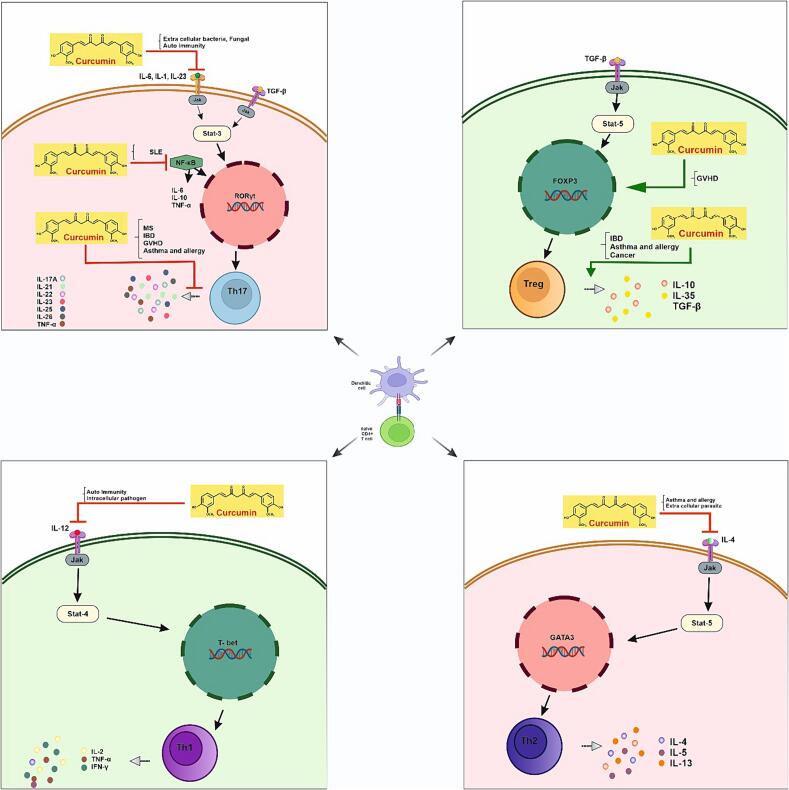
**The effect of curcumin on Th17/Treg balance in the context of autoimmune and inflammatory diseases.** Il-6 and TGF-b can stimulate naïve T cells to Th17. This pathway is inhibited by curcumin in RA. Curcumin can also inhibit the production of inflammatory cytokines in MS, IBD, GVHD, and asthma and allergy. Moreover, curcumin can inhibit the expression of Th17 by suppressing NF-kb. Furthermore, curcumin can induce FOXP3 to express Tregs in GVHD. It also can induce the production of anti-inflammatory cytokines from Trgs in IBD, cancer, and asthma and allergy. Curcumin, a polyphenol natural product derived from turmeric, possesses diverse pharmacological effects due to its interactions with various cells and molecules. Th17 cells play a crucial role in promoting immune responses against extracellular pathogens. Curcumin has been shown to significantly inhibit the proliferation of Th17 cells and reduce the production of inflammatory cytokines. This review aims to assess the effectiveness of curcumin and its underlying mechanisms in modulating Th17 cells.

In summary, curcumin has the ability to inhibit the differentiation and proliferation of Th17 cells by suppressing the expression of IL-23, IL-6, and IL-21. It also inhibits the release of pro-inflammatory cytokines such as IL-17 and TNF-α in Th17 cells, thereby reducing inflammation and the infiltration of inflammatory cells, particularly neutrophils, at the site of inflammation (Fig. 1). As a result, tissue degradation enzymes (elastase and collagenase) and pro-inflammatory molecules recruited by Th17 cells can be suppressed. Considering the role of Th17 cells and their associated cytokines in the development of RA, curcumin shows promise as a therapeutic agent for RA treatment.

### 8.3 Multiple sclerosis

Multiple sclerosis (MS) is a chronic autoimmune disease that affects the central nervous system (CNS), causing inflammation and damage to the protective covering of nerve fibers called myelin (ELBini-Dhouib et al., 2022). The exact cause of MS is not yet fully understood, but it is believed to be a combination of environmental factors and genetics (Xie et al., 2011, ELBini-Dhouib et al., 2022). Recent research suggests that T helper 17 (Th17) cells may play a key role in the development and progression of MS (Dolati et al., 2018, Xie et al., 2011). Th17 cells are a type of immune cell that produces pro-inflammatory cytokines, which can damage myelin and contribute to the symptoms of MS (Dolati et al., 2018). Studies have shown elevated levels of Th17 cells and related cytokines in the blood of MS patients, and targeting these cells with specific drugs may be effective in treating the disease (Dolati et al., 2018, ELBini-Dhouib et al., 2022).

Emerging evidence indicates that Th17 cells, compared to their Th1 counterparts, exhibit a heightened capacity to traverse the blood–brain barrier (BBB) in MS, thereby contributing to CNS inflammation. This enhanced migratory ability is facilitated by the expression of IL-17R and IL-22R on BBB endothelial cells (Kebir et al., 2007). Upon binding, IL-17 triggers ROS production via xanthine oxidase and NADPH oxidases, ultimately leading to increased BBB permeability and contractility through myosin light chain (MLC) phosphorylation (Huppert et al., 2010). This cascade disrupts tight junctions and endothelial monolayer integrity, creating avenues for Th17 cell infiltration and subsequent CNS inflammation (Kebir et al., 2007). Notably, MLCK inhibitors like ML-7 demonstrated the ability to preserve BBB integrity by mitigating IL-17-induced effects, further highlighting the role of IL-17 signaling in this process (Huppert et al., 2010). Therefore, researchers propose that targeting Th17 cells could be a promising approach for developing new treatments for MS (Dolati et al., 2018, ELBini-Dhouib et al., 2022). Intriguingly, curcumin presents a potential therapeutic approach for regulating Th17 cell migration in MS (Qureshi et al., 2018). However, further research is needed to fully understand the complex relationship between Th17 cells and MS and to develop targeted therapies that are safe and effective for patients.

### 8.4 Inflammatory bowel disease

Inflammatory Bowel Disease (IBD) is a chronic inflammatory disorder of the intestinal tract characterized by immune system dysregulation and uncontrolled inflammation (Guo et al., 2022, Hundorfean et al., 2012). Recent reports have suggested that T-helper 17 (Th17) cells play a vital role in the pathogenesis of IBD (Guo et al., 2022, Hundorfean et al., 2012).

Curcumin, a natural compound found in turmeric, has been found to possess potent anti-inflammatory properties and has been studied for its potential therapeutic effects on IBD (Guo et al., 2022). Wei et al. explored the therapeutic mechanisms of curcumin in a mouse model of UC induced by dextran sulfate sodium (DSS) (Wei et al., 2021). The study observed that curcumin modulated the balance between Tregs and Th17 cells by downregulating pro-inflammatory cytokines IL-6, IL-17, and IL-23, while simultaneously upregulating anti-inflammatory IL-10 in the colon. Guo et al. provided further support for curcumin's therapeutic potential in UC (Guo et al., 2022). Their study demonstrated that, in a DSS-induced colitis model, curcumin significantly increased the expression of Tregswhile decreasing the expression of Th17 cells. This suggests that curcumin modulates the Treg/Th17 balance, which plays a crucial role in UC pathogenesis. Additionally, curcumin upregulated the anti-inflammatory cytokine IL-10 and downregulated pro-inflammatory IL-17A, further supporting its beneficial effects. These findings, along with similar results from Xiao et al. who observed curcumin-mediated suppression of BATF, C‐Maf, and RORγt expression and induction of Foxp3 and Eomes, thereby inhibiting Th17 and increasing Tregs expression, respectively, highlight the potential of curcumin as a promising therapeutic strategy for UC (Xiao et al., 2022). Further research is necessary to fully elucidate the precise mechanisms underlying curcumin's therapeutic efficacy in UC and translate these findings into potential clinical applications.

### 8.5 Systemic lupus erythematosus

Systemic lupus erythematosus (SLE) is a chronic autoimmune disease characterized by the production of autoantibodies and dysfunction of the immune system (Momtazi-Borojeni et al., 2018, AlFadhli et al., 2016). The pathogenesis of SLE involves various immunological mechanisms, including the activation of T-helper cells, particularly Th17 cells (Momtazi-Borojeni et al., 2018, AlFadhli et al., 2016). Th17 cells play a crucial role in the development of inflammatory autoimmune diseases, including SLE (Momtazi-Borojeni et al., 2018, AlFadhli et al., 2016). Research has shown elevated levels of Th17 cells in SLE patients, leading to an increase in pro-inflammatory cytokines (Momtazi-Borojeni et al., 2018). Therefore, targeting Th17 cells may be a promising approach for treating SLE (Momtazi-Borojeni et al., 2018).

Curcumin therapeutic potential arises from its ability to target multiple key signaling pathways involved in inflammatory responses. One primary mechanism involves inhibiting the IκB kinase β (IKKβ) subunit of the NF-κB pathway, thereby suppressing downstream production of pro-inflammatory cytokines such as IL-6 and TNF-α by T cells (Ravi et al., 2022) (Fig. 1). This action effectively dampens the inflammatory cascade and protects tissues from damage. Furthermore, curcumin exhibits regulatory effects on TLRs, critical players in recognizing self-antigens and contributing to autoimmune pathogenesis. By modulating TLR activity, curcumin can mitigate inappropriate immune activation and subsequent tissue inflammation (Ravi et al., 2022).

Clinical research adds further credence to curcumin's potential. A study demonstrated a significant reduction in Th17 cell populations within the CD4 + T cell population in SLE patients treated with curcumin, highlighting its capacity to directly influence inflammatory effector cell populations (Handono et al., 2015).

In conclusion, the diverse mechanisms by which curcumin targets key inflammatory pathways, coupled with encouraging clinical observations, suggest its strong potential as a therapeutic agent for managing SLE by modulating the immune response to self-antigens (143, 144). However, further research is necessary to fully elucidate its long-term effects and optimize its application in clinical settings.

### 8.6 Autoimmune myasthenia gravis

Curcumin has been shown to promote a shift in the balance of Th1/Th17 toward Th2/Treg in the context of experimental autoimmune myasthenia gravis (Wang et al., 2016).

## 9 The effect of curcumin on Th17 in inflammatory diseases

### 9.1 Organ transplantation

Organ transplantation relies on factors such as dendritic cells and Treg cells for tolerance. Curcumin can inhibit the maturation and differentiation of dendritic cells and promote a tolerogenic phenotype that enhances the suppressive function of CD4 + CD25 + FoxP3 + Treg cells. Dendritic cells treated with curcumin demonstrate reduced expression of co-stimulatory molecules, IL-12 mRNA, and protein secretion. Upon T cell stimulation, intracellular IFN-gamma expression and the allostimulatory capacity of dendritic cells are significantly decreased. Curcumin-treated dendritic cells further decrease alloproliferative capacity and induce the development of CD4 + CD25 + FoxP3 + Treg cells (Rogers et al., 2010).

In a murine model of acute graft-versus-host disease (GVHD), curcumin can improve disease complications by reducing the levels of Th1 and Th17 cells and suppressing the production of IL-17 and IFN-gamma (Park et al., 2013). These findings collectively indicate that curcumin can enhance the function and percentage of Treg populations, demonstrating a protective impact on GVHD.

### 9.2 Asthma and allergy

Multiple studies have investigated the modulatory effects of curcumin on Th17 in various allergic diseases. One study conducted by Chen et al. demonstrated the efficacy of curcumin and THC (an active metabolite of curcumin) in inhibiting the differentiation of the Th17 subset and the production of its signature cytokine, IL-17 (Heidari et al., 2022). THC has also been shown to have a significant therapeutic impact in asthmatic mice, primarily through the suppression of Th17 cells and associated cytokines, such as IL-17A (Wu et al., 2020). Ma et al. (Ma et al., 2013) revealed that curcumin effectively increases the proportion of Treg cells while significantly reducing Th17 cell differentiation in cases of allergic asthma. As a result, curcumin's regulatory effect on the imbalance between Th17 and Treg cells leads to a reduction in elevated levels of IL-17A. Conversely, curcumin can stimulate IL-10 production through Treg cells in lung tissue (Ma et al., 2013). Similarly, Yuan et al. reported that FLLL31, a novel derivative of curcumin, exhibited inhibitory effects on Th17 cell differentiation in primary in-vitro experiments. At the molecular level, FLLL31 suppressed STAT3 phosphorylation, a key transcription factor involved in Th17 differentiation, thereby impeding the production of IL-17 in vivo (Yuan et al., 2014) (Table 1). These findings collectively provide compelling evidence to suggest that curcumin may hold therapeutic promise in the treatment or management of allergic diseases through its modulatory effects on Th17 and Treg cells.

### 9.3 Cancers

Effector and memory T cells play a role in targeting and inhibiting the escape of tumor cells from the immune system (Yu et al., 2007, Talmadge and Gabrilovich, 2013, Mao et al., 2014). In tumor-bearing hosts, curcumin can inhibit T-cell loss, increase the proportion of effector and memory T cells, and prevent the inhibition of T-cell proliferation (Bhattacharyya et al., 2010). Furthermore, tumors contribute to the up-regulation of Treg cell populations and the induction of immunosuppressive cytokine production, including TGF-β and IL-10 (Zou et al., 2018). Curcumin can alter the tumor microenvironment, leading to enhanced cytotoxicity of CD8 + T cells against tumors. Additionally, inhibiting various immunosuppressive agents promotes the function and accumulation of T cells (Chang et al., 2012).

## 10 Conclusion and future perpectives

The immune system is a complex network of cells and molecules that work together to protect the body against infections and diseases. The balance between Treg cells and Th17 is crucial for maintaining a healthy immune response, and any alterations in this balance can lead to chronic inflammatory disorders. Curcumin, an active compound found in turmeric, exhibits potent immunomodulatory effects, making it a promising therapeutic agent for various inflammatory and autoimmune disorders. Recent studies have focused on the ability of curcumin to modulate Th17-mediated immune responses, which play a critical role in the development of several autoimmune diseases. Curcumin has been shown to inhibit the proliferation and differentiation of Th17 cells and reduce the production of specific pro-inflammatory cytokines such as IL-17, IL-6, and TNF-α. Additionally, curcumin has been found to increase the production of Treg cells, which suppress excessive immune responses and maintain immune homeostasis. Overall, curcumin's ability to target the Th17 axis opens exciting avenues for developing novel therapeutic strategies for autoimmune and inflammatory diseases.

While curcumin exhibits promising potential in modulating Th17-mediated inflammation, several key research gaps hinder its full therapeutic translation. A deeper understanding of its precise molecular mechanisms, particularly interactions with key Th17 regulatory factors, is essential. Additionally, poor bioavailability necessitates exploring formulations and delivery systems for enhanced absorption and targeted tissue delivery. Clinical trials with standardized protocols and long-term follow-up are crucial to evaluate efficacy and safety across diverse disease contexts. Investigating synergistic effects with other therapeutic agents and implementing personalized medicine approaches based on individual response variations hold significant promise for improving efficacy and minimizing side effects. Furthermore, research efforts focused on developing novel curcumin derivatives with improved bioavailability and targeted activity, tailoring curcumin-based interventions to specific disease contexts, and exploring its broader applications in other inflammatory conditions and even cancer offer exciting future perspectives. By addressing these limitations and pursuing these promising approaches, we can unlock the full potential of curcumin in combating Th17-mediated inflammation and developing novel therapeutic strategies for various autoimmune and inflammatory diseases.

## Funding

None

## CRediT authorship contribution statement

**Ehsan Ghoushi:** Writing – original draft. **Mohadeseh Poudineh:** Writing – original draft, Conceptualization. **Negin Parsamanesh:** Writing – review & editing. **Tannaz Jamialahmadi:** Writing – review & editing. **Amirhossein Sahebkar:** Writing – review & editing, Conceptualization.

## Declaration of competing interest

The authors declare that they have no known competing financial interests or personal relationships that could have appeared to influence the work reported in this paper.

## Data Availability

No data was used for the research described in the article.
